# Confirmatory factor analysis of Clinical Outcomes in Routine Evaluation (CORE-OM) used as a measure of emotional distress in people with tinnitus

**DOI:** 10.1186/s12955-016-0524-5

**Published:** 2016-09-06

**Authors:** L. Handscomb, D. A. Hall, D. J. Hoare, G. W. Shorter

**Affiliations:** 1 National Institute for Health Research Nottingham Hearing Biomedical Research Unit, Otology and Hearing Group, Division of Clinical Neuroscience, University of Nottigham, 113 The Ropewalk, Nottingham, NG1 5DU UK; 2 UCL Ear Institute, 332 Gray’s Inn Road, London, WC1X 8EE UK; 3 Alcohol and Public Health Team, Health and Social Care Institute, Teesside University, Middlesbrough, TS1 3BA UK; 4 Northern Ireland Association for Mental Health, 80 University St, Belfast, BT7 1HE UK; 5 Psychotraumatology, Mental Health, and Suicidal Behaviour Group, Psychology Research Institute, Ulster University, Coleraine, BT52 1SA UK

**Keywords:** CORE-OM, Tinnitus, Emotional distress, Confirmatory factor analysis

## Abstract

**Background:**

People with troublesome tinnitus often experience emotional distress. Therefore a psychometrically sound instrument which can evaluate levels of distress and change over time is necessary to understand this experience. Clinical Outcomes in Routine Evaluation (CORE-OM) is a measure of emotional distress which has been widely used in mental health research. Although originally designed as a 4-factor questionnaire, factor analyses have not supported this structure and a number of alternative factor structures have been proposed in different samples. The aims of this study were to test the factor structure of the CORE-OM using a large representative tinnitus sample and to use it to investigate levels of emotional distress amongst people with a range of tinnitus experience.

**Methods:**

The CORE-OM was completed by 342 people experiencing tinnitus who self-rated their tinnitus on a 5-point scale from ‘not a problem’ to ‘a very big problem’. Confirmatory factor analysis was used to test all ten factor models which have been previously derived across a range of population samples. Model fit was assessed using fit criterion and theoretical considerations. Mean scores on the full questionnaire and its subscales were compared between tinnitus problem categories using one-way ANOVA.

**Results:**

The best fitting model included 33 of the 34 original items and was divided into three factors: negatively worded items, positively worded items and risk. The full questionnaire and each factor were found to have good internal consistency and factor loadings were high. There was a statistically significant difference in total CORE-OM scores across the five tinnitus problem categories. However there was no significant difference between those who rated their tinnitus ‘not a problem’, and ‘a small problem’ or ‘a moderate problem.’

**Conclusion:**

This study found a 3-factor structure for the CORE-OM to be a good fit for a tinnitus population. It also found evidence of a relationship between emotional distress as measured by CORE-OM and perception of tinnitus as a problem. Its use in tinnitus clinics is to be recommended, particularly when emotional distress is a target of therapy.

**Electronic supplementary material:**

The online version of this article (doi:10.1186/s12955-016-0524-5) contains supplementary material, which is available to authorized users.

## Background

Many people who experience tinnitus also experience emotional distress [1–4]. Emotional distress has been found to be significantly greater amongst those who rate their tinnitus as more troublesome in both tinnitus clinic patients [5], and members of voluntary tinnitus support organisations [6]. Emotional distress is likely to be one of the factors that makes tinnitus a major problem for some people, negatively affecting their quality of life [7]. A strong correlation has been shown between measures of anxiety and depression and catastrophic thinking about tinnitus [8]. It has been proposed that a negative feedback loop may exist in people who are troubled by tinnitus, with negative thoughts increasing emotional distress and emotional distress in turn increasing attention towards and negative thoughts about tinnitus [7]. There is a greater association between symptoms of generalised anxiety and depression and tinnitus distress than there is between these symptoms and perceived tinnitus loudness [6], and negative emotional state has been shown to be a partial mediator between tinnitus loudness and tinnitus distress [9]. Conversely, tinnitus may be perceived as loud but not annoying by people who are not depressed [10]. These findings suggest that emotional distress plays an important role in determining people’s tinnitus experience, independently from loudness of sound.

Clinical guidelines recommend that symptoms of anxiety and depression should be routinely assessed as part of history taking with tinnitus patients [11] so that onward referrals to mental health services can be made when needed. In addition, many forms of therapy for tinnitus, particularly cognitive behavioural therapy, relaxation and mindfulness meditation, are designed to help people better live with their tinnitus, in part by reducing the emotional distress that surrounds it. A reliable means of measuring emotional distress before and after treatment is therefore important for fully evaluating the clinical efficacy of tinnitus therapies.

A number of investigations of psychological therapy for people with tinnitus have reported reductions in general emotional distress post treatment as well as improved scores on tinnitus-specific measures (for example [12–15]) while others have reported significant changes in tinnitus distress without corresponding changes in measures of general emotional distress [16, 17]. There are a number of possible reasons for this discrepancy, one of which of which is that the majority of such studies have confined themselves to the measurement of specific symptoms of generalised anxiety and depression, using measures such as the Hospital Anxiety and Depression Scale (HADS; [18]), the Beck Depression Inventory (BDI; [19]), or the Beck Anxiety Inventory (BAI; [20]). While a significant proportion of tinnitus patients- approximately 40 %- meet the criteria for a concurrent diagnosis of generalised anxiety and/or clinical depression [21, 22] many do not, and mean scores on measures of generalised anxiety and depression at the start of treatment are often fairly low [16, 17]. Such measures may not be sufficiently sensitive to capture benefits of therapy to emotional wellbeing in less severely affected individuals.

A measure of general emotional distress which is deliberately not specific to any particular diagnosis is Clinical Outcomes in Routine Evaluation (CORE-OM; [23]). It is designed for assessment and treatment evaluation and contains a mix of high and low intensity items related to overall emotional wellbeing. “I have been able to do most things I needed to” and “I have felt panic or terror” are examples of low and high intensity items respectively. Convergent validity with older measures of emotional distress has been shown to be high, but indicative of some distinction between the concepts measured. Correlation with the BDI was *r* = 0.85, with the BAI *r* = 0.65 [24], with HADS anxiety *r* = 0.74 and with HADS depression *r* = 0.66 [25]. The CORE-OM has been widely and successfully used to measure emotional distress across a range of conditions such as chronic anxiety [26], depression [27], and eating disorders [28]. The items which make up the CORE-OM assess overall mental wellbeing by enquiring about both mental processes (e.g. “unwanted images or memories have been distressing me”) and feelings (e.g. “I have felt tense, anxious or nervous”.)

The CORE-OM was originally organised into four domains: well-being (4 items), social functioning (12 items), problems/symptoms (12 items), and risk (6 items), through discussion within the development team and consultation with therapists, researchers, and service users [23]. The authors suggested domain scores could be calculated in addition to the overall score. While the original four domains were all shown to have a high degree of internal consistency α = 0.75-0.94 [24], factor analysis was not used to confirm the relationship between variables. Subsequent factor analyses in differing datasets have supported a range of factor structures with the original four domains not supported except in one study to date. Uji et al. [25] who conducted an analysis of the Japanese version of the CORE-OM found the 4 factor structure to be a reasonable fit (RMSEA = 0.062, GFI = 0.867).

Evans et al. [24] used exploratory principle components analysis to analyse scores from 890 mental health clinic patients from several UK sites and 1106 students without diagnosed mental health problems. For both groups, a three factor solution best fit the data, comprising positively worded items (*n* = 8), negatively worded items (*n* = 19), and items indicating risk to oneself or others (*n* = 6). Factor loadings were moderate to high, but item 8 (“I have been troubled by aches, pains or other physical problems”) was rejected for the student group because it did not load onto any factor. Barkham et al. [29] also reported a three factor solution to their data from two samples of older adults (aged 65–97); of which 118 were adults attending mental health services, and 214 were adults from the general population. However, the items were differently distributed across the factors compared with Evans et al. [24] and five items were excluded as they had low factor loadings <0.4. The authors suggest these differences may be due to the increased likelihood that older responders are limited in life by physical problems. Older people are also more likely to live alone and to have lost some sources of social support.

Bedford et al. [30] also conducted principal-components analysis using data from 543 people referred to a psychological therapy service in the UK. They found a two- factor solution to be optimal, composed of 23 ‘psychological distress’ items and five ‘risk’ items. The remaining six of the 34 original items were rejected for having insufficient factor loadings. These authors conducted Mokken scaling of the CORE-OM and suggested the number of items in both factors could be reduced further without loss of sensitivity. Murray et al. [31] also carried out Mokken scaling using data from 360 students who had been referred to a student counselling service at a UK university. This process resulted in a ten-item, single-factor measure.

Two studies have examined more complex structures. A detailed study involving CORE-OM data from 2140 patients referred to psychotherapy and counselling services across the UK was conducted by Lyne et al. [32]. They used Confirmatory Factor Analysis to test alternate models, which they then compared to each other. The four factor model proposed by the original authors was found to be a fairly poor fit (RMSEA = 0.074, CFI = 0.84). In addition, two risk items (involving risk to others) were found to be poorly related to the rest of the risk factor and were excluded from subsequent analyses. The three factor model supported by the work of Evans et al. [24] was also a less- than- optimal fit (RMSEA = 0.070, CFI = 0.87). The best fit (RMSEA = 0.051, CFI = 0.93) was achieved by a higher order model, in which there was one first-order ‘general psychological distress’ factor with four subordinate factors (the four domains originally proposed) and an additional two subordinate method factors (positive and negative). However, the authors note there is a high degree of correlation between the three non-risk domains (ρ = 0.75-0.79), and suggest for practical use, treating the CORE-OM as a two factor measure (general psychological distress and risk to self) is adequate.

The psychometric properties of a Norwegian translation of the CORE-OM were explored using data from 527 mental health clinic patients and 464 members of the general population [33]. Using confirmatory factor analysis, these researchers again found the original four factor structure to be a fairly poor fit (RMSEA = 0.080, CFI = 0.94) and they also note a moderate to high correlation between the three non-risk factors (ρ = 0.62-0.74), suggesting they may not be conceptually distinct. Similarly to Lyne et al. [32], they found a higher order factor model to be the best fit (RMSEA = 0.057, CFI = 0.97) with a general psychological distress factor and subordinate factors consisting of the four original domains. Adding positive and negative items to their model did not improve fit, but they did not test a three factor model consisting of positive, negative and risk factors only. They conclude by recommending that CORE-OM be treated as a two factor measure (general psychological distress and risk) because this makes it a simple way to identify those at risk and measure the effects of therapy.

In summary, 1, 2, 3, and 4 factor structures have been proposed for the CORE-OM. The various patterns of item distribution and omission of different items make it difficult to understand and compare findings across studies and in practice. Whilst the CORE-OM is used in clinical practice across a range of health conditions, there are no studies analysing the psychometric properties of this questionnaire in people with tinnitus. This sample is important; it represents those who may suffer distress as a consequence of a body-related condition. The majority of analyses to date have assessed the factor structure in samples drawn from mental health clinics or those in non-clinical samples (including students or people drawn from the general population) and it is unclear whether any of these structures will fit a tinnitus population. Knowledge of the validity and optimal factor structure of the CORE-OM amongst people with tinnitus will be of benefit to clinicians looking for a reliable means to assess the mental health of their tinnitus clinic patients and measure the effects of treatment.

The current study was carried out as part of a larger investigation of the components of tinnitus distress. The primary aim of this study was to explore the psychometric properties of the CORE-OM in a sample of people with tinnitus through confirmatory factor analysis of all the previously proposed structures, in order to determine the degree to which this instrument measures what it claims in this particular population. Higher order factor models were not tested as these are not practical for clinical use. We planned to use exploratory factor analyses to determine a meaningful factor structure should none of these models be of satisfactory fit to the data. The secondary aim was to investigate whether general emotional distress (as measured by the CORE-OM) is greater amongst those who rate their tinnitus as more of a problem.

## Methods

### Participants

The data analysed in this study were obtained from 342 adults experiencing tinnitus who were recruited from NIHR Nottingham Hearing Biomedical Research Unit (BRU) volunteer database, the British Tinnitus Association (BTA) member magazine, ‘Quiet’, and social media sites run by the BTA and the charity Hearing Link. Participants in the survey completed the CORE-OM along with eight other questionnaires online (*n* = 323; 94.4 %) or in paper format (*n* = 19; 5.6 %). For a full list of questionnaires used please see [34]. Two individuals were listwise deleted from the dataset as they did not answer all the questions on the CORE-OM questionnaire, leading to an effective sample size of 340. The mean age of the sample was 55.0 years old (standard deviation = 13.3 years), 185 (54.3 %) were male. To describe the spread of tinnitus experience across individuals with differing perceptions of the problematic nature of their tinnitus, participants were stratified by the answer to the question: “How much of a problem is your tinnitus?” which was asked at the time of recruitment. This is in line with previous research by Zeman et al. [35] who used a similar system. They found volunteers on the Tinnitus Research Initiative database proportionally fell into five categories, with more people reporting moderate levels of distress than either very low or very high levels. These figures were used as a guide to ensure that different degrees of tinnitus suffering were fairly represented in the current study, and recruitment to each category was stopped once the target number had been reached. Thirty five (10.2 %) participants were in the ‘no problem’ category, 85 (24.9 %) in the ‘small problem’ category, 102 (29.8 %) in the ‘moderate problem category’, 83 (24.3 %) in the ‘big problem’ category and 37 (10.8 %) in the ‘very big problem’ category. The mean duration of tinnitus was 14.0 years (SD = 13.7; range = 3 months- 69 years).

### Ethical approval and consent

Ethical approval was granted by the University of Nottingham’s School of Medicine research ethics committee (reference: G13022014 School of Medicine NIHR NRU Hearing). Consent to participate was obtained by means of an online or postal consent form.

#### Questionnaire

The CORE-OM [23] is a 34-item self-report measure of psychological distress. Respondents are asked to respond to questions about their emotions and actions during the past week on a five point Likert scale (from ‘not at all’ to ‘most of or all the time.’) There are a mix of positive and negative items (positive items are reverse-scored) and the total score ranges from 0–136. A cut-off score of 34 has been suggested as the minimum indicator of mild psychological distress within the clinical range [36]. Initial validation of the full CORE-OM [24] found it to have very high internal consistency (α = 0.94) and convergent validity with 10 other measures of psychological distress (ρ = 0.55-0.88).

#### Analysis

Mean scores were calculated and one- way analysis of variance was conducted to identify whether there were statistically significant differences in mean scores between the five tinnitus problem categories for the original factor structure of the CORE-OM. Post-hoc tests were used to identify significant differences between specific categories.

Following this, a series of confirmatory factor models were specified and estimated using the robust weighted least squares (WLSMV) estimator accounting for the categorical nature of response data. Model fit was based on a consensus of fit criteria and theoretical considerations. The fit criteria include the Root Mean Square Error of Approximation and 90 % confidence intervals (RMSEA; [37]. A value of less than 0.05 represents good fit, errors of approximation of up to 0.08 are considered an acceptable absolute fit [38], and a RMSEA of between 0.08 and 0.1 is considered a mediocre fit [39]. Other fit indices include the chi-square statistic, with a non-significant chi-square representing a well- fitting model, although it may be affected by large sample sizes [40]. The Comparative Fit Index (CFI; [41]) and Tucker Lewis Index (TFI; [42]) are both comparative fit indices. A value of >0.95 is considered to indicate a good fit [43].

Ten models were specified and estimated in the analysis. Model I, the original classification, was a four factor model with items 4, 14, 17, and 31 loading on factor one: ‘subjective wellbeing’, items 1, 3, 7, 10, 12, 19, 21, 25, 26, 29, 32, and 33 loading on factor two: ‘functioning’, items 2, 5, 8, 11, 13, 15, 18, 20, 23, 27, 28, and 30 loading on factor three: ‘problems/ symptoms’ and items 6, 9, 16, 22, 24, and 34 loading on factor four: ‘risk’.

Model II was a three factor model identified and recommended for ‘further exploration’ by Evans et al. [24] with items 1, 2, 5, 8, 10, 11, 13–15, 17, 18, 20, 23, 25–30, and 33 loading onto factor one: ‘negatively worded’, items 3, 4, 7, 12, 19, 21, 31, and 32 loading onto factor two: ‘positively worded’ and items 6, 9, 16, 22, 24, and 34 loading onto factor three: ‘risk’. Although item 8 did not load onto any factor for Evans et al’s non-clinical population, it had quite a high ‘negative’ loading for their clinical population and was retained in this study as the authors do not recommend its deletion when exploring this structure further.

Model III was identical to model II except that items 6 and 22 were omitted from the risk factor in accordance with Lyne et al. [32].

Model IV was the three factor model specified by Barkham et al. [29] for their clinical population (which they consider to be of principal interest) with items 1, 2, 5, 11, 13, 14, 15, 17, 20, 23, and 27 loading onto factor one: ‘negatively worded', items 3, 4, 7, 12, 19, 21, 31 and 32 loading onto factor 2, ‘positively worded’, and items 6, 9, 16, 22, 24, 25, 26, 29, 33, and 34 loading onto factor 3, ‘risk’.

Model V was a two factor model recommended for use by Skre et al. [33] with items 1–5, 7–21, 23, and 25–33 loading on factor one: ‘general psychological distress’ and items 6, 9, 16, 22, 24, and 34 loading on factor two: ‘risk.’ Model VI was identical to model V but excluded items 6 and 22 in accordance with Lyne et al. [32]. Model VII was also a two factor model similar to model V, but excluding items 8, 24, 25, 28, 29, and 33 in accordance with Bedford et al. [30].

Model VIII was a single factor model containing all 34 items. Although this model has not been found to be optimal in any previous studies [30, 33], it was tested for the sake of completeness. Model IX was the 6-item Mokken scale recommended by Bedford et al. [30] and model X was the 10-item Mokken scale recommended by Murray et al. [31].

The complex, higher-order models proposed by Lyne et al. [32] and Skre et al. [33] were not tested in this study. Although they were shown to be well fitting, these models are impractical for clinical use because they do not allow individual factor scores to be calculated and the purpose of this study is to offer guidance on how the CORE-OM might best be used by clinicians who wish to obtain a score and/ or sub-scores reflective of emotional distress amongst their tinnitus patients.

Following this, a further one-way analysis of variance was carried out to compare scores between problem categories using the item distribution of the best-fitting model identified in this study. Post-hoc tests were used to identify significant differences between specific categories.

Reliability of the whole scale and of each subscale in the final model was also tested and divergent validity of the full CORE-OM with two measures of tinnitus distress; the Tinnitus Reaction Questionnaire [44] and the emotional subscale of the Tinnitus Functional Index [45].

## Results

### Mean scores

The overall mean score on the CORE-OM questionnaire was 32.96 (SD = 22.33). Those who rated their tinnitus as less problematic had lower scores on the CORE-OM questionnaire. Table 1 shows mean scores and standard deviations for each ‘problem category’, for the full questionnaire, and for each of the original four CORE-OM domains.

**Table 1:** Mean (SD) CORE-OM full questionnaire and domain scores, overall and grouped by tinnitus problem category

	Self-reported tinnitus problem category						
	No problem	Small problem	Moderate problem	Big problem	Very big problem	Overall	Max total
Global Emotional Distress	21.97 (17.06)	22.17 (15.61)	29.29 (16.70)	41.89 (22.27)	59.14 (25.94)	32.96 (22.33)	136
Subjective wellbeing	3.56 (3.28)	3.49 (3.06)	4.81 (2.90)	6.60 (3.51)	9.54 (3.55)	5.29 (3.71)	16
Functioning	9.40 (6.88)	10.12 (6.05)	11.90 (6.56)	15.60 (8.73)	21.60 (9.71)	13.15 (8.26)	48
Problems	8.80 (8.25)	8.24 (7.77)	12.25 (8.18)	18.40 (9.56)	25.83 (11.66)	13.81 (10.45)	48
Risk	0.31 (0.87)	0.32 (1.24)	0.34 (0.88)	1.30 (3.06)	2.89 (4.29)	0.84 (2.36)	24

A one-way between groups analysis of variance was conducted to explore the effect of problem category on mean score on the CORE-OM. There was a statistically significant difference in CORE-OM scores for the 5 problem categories: F (4, 335) = 31.97, *p* < 0.01. Post-hoc comparisons using the Tamhane T2 test indicated the mean score for the no problem group (M = 21.97, SD = 17.07) was not significantly different from the mean score for the small problem group (M = 22.17, SD = 15.62) or for the moderate problem group (M = 29.29, SD = 16.70).

Examination of the correlation matrices indicated that for item 3 (“I have felt I have someone to turn to for support when needed”) correlation coefficients were very low, and mostly negative in relation to other items (as positively worded items are reverse-scored, a positive correlation should be expected). Factor analyses were run on datasets with and without item 3; this item did not load onto any factor in any of the two, three, or four factor solutions. As such the results are given below without item 3.

### Confirmatory factor analysis

The fit of all 10 factor analyses is shown in Additional file 1: Table S1. For the original four factor structure (Model I), the chi-square was large relative to the degrees of freedom, but other fit criteria appeared acceptable. However, the correlation between factors one and three was greater than 1 (ρ = 1.007). This suggests the original four factor solution is not a good fit to the data.

Model II comprising negative, positive and risk factors provided the best fit. In Model III, omitting items 6 and 22 from the risk factor resulted in a much poorer fit. Model IV, in which the items were differently distributed between the negative, positive, and risk factors, was a very poor fit. Model V, the two factor structure recommended by Skre et al. [33] yielded a reasonable fit. In model VI, omitting items 6 and 22 from the risk factor again resulted in a poorer fit. Model VII, the 28-item, two factor model, was also a worse fit than Model V. Model VIII, the single-factor model including all items, did not meet good fit criteria. Model IX, the shortened 6-item scale, was a mediocre fit, while model X, the 10-item scale, was an acceptable fit to the data. The best fitting model overall was model II. For all items, factor loadings were positive, moderately to very high, and statistically significant. There was a high degree of positive correlation between all three factors.

Factor scores for each of the three factors in the best fitting model (Model II) are shown in Table 2, overall and divided by tinnitus problem category.

**Table 2:** Factor scores for negative, positive, and risk factors by tinnitus problem category

	Self-reported tinnitus problem category						
Factor	No Problem	Small problem	Moderate Problem	Big problem	Very big problem	Overall	Max total
Negative	12.49 (13.67)	11.95 (11.85)	17.86 (12.96)	27.01 (15.67)	39.12 (18.50)	20.29 (16.45)	80
Positive	6.80 (5.61)	7.17 (5.30)	8.57 (4.80)	11.15 (5.75)	14.95 (5.56)	9.36 (5.86)	28
Risk	0.31 (0.87)	0.32 (1.24)	0.34 (0.88)	1.30 (3.06)	2.89 (4.23)	0.84 (2.36)	24

A one-way between groups analysis of variance was conducted to explore the effect of problem category on mean score on the three factors of the best-fitting version of CORE-OM. For each factor, there was a statistically significant difference at the p < 0.05 level in CORE-OM scores for the five problem categories (negative factor: F (4, 335) = 31.47, p < 0.01; positive factor: F (4, 337) = 18.68, *p* < 0.01; risk factor: F (4,337) = 11.75, *p* < 0.01). Post-hoc comparisons using the Tamhane T2 test indicated that, on the negative factor, the mean score for the no problem group (M = 12.49, SD = 13.67) was not significantly different from the mean score for the small (M = 11.95, SD = 11.85) or moderate problem (M = 17.86, SD = 12.96) groups but all other differences were statistically significant. On the risk factor, the only significant differences between mean scores were between the very big problem group (M = 2.89, SD = 4.23) and the no (M = 0.31, SD = 0.87), small (M = 0.32, SD = 1.24) and moderate (M = 0.34, SD = 0.88) problem groups. On the positive factor, post hoc comparisons using Bonferroni correction indicated that there was no statistically significant difference between the no problem (M = 6.80, SD = 5.61) and small problem (M = 7.17, SD = 5.30) and moderate problem (M = 8.57, SD = 4.80) groups nor between the small problem and moderate problem groups, but all other differences were statistically significant.

### Reliability

Reliability of the subscales for the best fitting model (Model II) was calculated. For the negative scale α = 0.95, for the positive scale α = 0.83, and for the risk scale α = 0.80, representing high reliability. For the global score (excluding item 3) α = 0.95.

### Divergent validity

Correlation between the CORE-OM and two measures of tinnitus distress was calculated using Spearman’s rho. With the Tinnitus Reaction Questionnaire ρ = 0.73, p < 0.001, and with the emotional subscale of the Tinnitus Functional Index ρ = 0.67, p < 0.001.

## Discussion

The findings of this study indicate that the CORE-OM is a valid and useful way to measure emotional distress in people with tinnitus. They also demonstrate a relationship between emotional distress and how problematic a person perceives their tinnitus to be.

In line with several previous studies, this analysis found the original 4-domain structure of the CORE-OM did not fit the data well. The CORE-OM continues to be considered as a measure with four subscales in many trials involving psychological therapy [e.g. 26, 46] but individual domain scores and mapping change using these scores may not in fact be meaningful, particularly in a tinnitus sample.

The best fit to our tinnitus population data was to a model reported by Evans et al. [24] which comprises three domains: positive well-being, negative feelings, and risk (to self and others). Although the risk factor was highly correlated with both the other factors, it seems important to take note of risk scores separately as a high score indicates significant risk of self-harm or violence and therefore a need for specialist help. Although self-harm amongst tinnitus patients is rare, cases in which tinnitus has been given as a reason for suicide have been documented [47], and clinicians should be alert to this possibility during their consultations. In the current study, responses to item 16 (“I made plans to end my life”) were used to identify a small number of individual respondents at potential risk of suicide. They were emailed by the principal researcher with the offer of a letter to their general practitioner highlighting the extent of their challenges. Two participants responded to say they were grateful for this support. Negative and positive factors were also highly correlated with each other, but collapsing these two subscales into one (Model V) resulted in a worse fit. A high degree of correlation might be expected, as some positive and negative items are mutually exclusive (it is unlikely that somebody would feel optimistic about his future while simultaneously thinking he was better off dead). However, there are some positive statements which might be agreed with even by somebody with predominantly negative feelings (e.g. “I have felt warmth or affection for someone”). Obtaining distinct positive and negative scores allows for evaluation of whether smaller improvements in overall emotional wellbeing come about chiefly through increases in positive feelings or decreases in negative feelings, or both. This in turn could help to inform the content of therapy. Nevertheless, some caution should be used when interpreting responses to positive and negative subscales as a body of research has shown that distinct positive and negative subscales may emerge due to wording preferences more than to item content [48, 49].

Although previous studies have excluded various different items on the grounds of low factor loadings, in the current study item 3 was the only one with a low factor loading. The raw data for this item were scrutinized and it appears that many people who scored low on the questionnaire overall (indicating good psychological wellbeing) scored high on this item. This is the first positively worded item in the questionnaire and reads: “I have felt I have someone to turn to for support when needed”. It is possible that this item was misread by many participants as a negative statement. An alternative possibility is that people who are not psychologically distressed do not feel in need of support and so did not find this item applicable.

Our findings indicate that a much-shortened, 10-item version of the CORE-OM was also an acceptable fit in this sample. This raises the interesting possibility that a brief version of the CORE-OM may well be appropriate for use in tinnitus clinics, with obvious advantages in terms of time and burden on patients who may also be completing additional tinnitus-specific questionnaires. Item 16 (“I made plans to end my life”) is retained, so suicide risk is still flagged. However, further testing of this brief version is needed before it can be recommended with confidence.

The tendency for people who rate their tinnitus as a ‘very big problem’ to score high on the CORE-OM supports the theory that emotional distress is associated with a worse experience of tinnitus. Information about other current medical conditions was not collected in the survey so it is not known whether these people were more likely experience other health problems, which might have affected their CORE-OM scores. Interestingly, the mean duration of tinnitus (M = 9.0 years, SD = 13.3) was shorter for the ‘very big problem’ group’ than for any of the other problem groups, which raises the possibility that emotional distress decreases over time.

It is notable there was no significant difference in mean total scores between ‘no problem’ and ‘moderate problem’ groups. There were some relatively high scores on the CORE-OM within the ‘no problem’ group, indicating that some people who do not find their tinnitus to be a problem may be emotionally troubled by other things such as stressful life events. There were also a number of very low (<20) CORE-OM scores in the ‘moderate problem’ group suggesting that, for some, bothersome tinnitus does not greatly affect overall wellbeing. The same pattern was not found when comparing mean scores between groups on the two measures of tinnitus-related distress used (the TRQ and the TFI emotional subscale); for these measures there was a statistically significant difference in mean scores between all problem groups. In addition, the correlation between these two measures of tinnitus-related distress and the CORE-OM, while strong, suggests some discrepancy between them. Taken together, these findings support the idea of using a measure of general emotional distress as well as one of tinnitus-related distress in clinics. In particular, this may help to establish whether effects of therapy are due to reducing tinnitus distress specifically or improving emotional wellbeing more generally, which has implications for the development of treatment. It may be, for example, that targeting thoughts about tinnitus specifically is more effective than targeting emotional wellbeing more generally.

As this study involved only volunteers with tinnitus from the general population, the findings cannot be assumed to apply to tinnitus clinic patients. Although some participants were seeking or had sought help for their tinnitus, some had not, and in this way they are different from an exclusively clinical population. Future research could usefully analyse and compare CORE-OM responses from patients attending tinnitus clinic appointments in a variety of settings such as audiology departments, psychology services, or community-based clinics.

## Conclusions

This study has demonstrated that a 33-item, three factor model of the CORE-OM is the best fit to data obtained from a sample of people in the general population with tinnitus. The removal of item 3 improved the fit of this data, but there is no theoretical reason why the statement “I have felt I have someone to turn to for support when needed” should not be applicable to tinnitus patients and it may be wise to retain it in future studies. The findings also provide some evidence of a link between emotional distress and perception of tinnitus as a problem, although the relationship is not straightforward.

The CORE-OM can be used to measure positive and negative emotions and to identify at-risk individuals. Although it has not been tested on a clinical tinnitus population, this analysis does raise the possibility that it could be used as a valid measure of emotional distress in people with tinnitus. Given the contribution of emotional distress to people’s tinnitus experience and the importance of reducing it in therapy, the addition of the CORE-OM to the list of outcome measures used in clinics should be carefully considered.

## Additional file

Additional file 1: Table S1. Fit indices, factor loadings and correlations between factors for 10 models of the CORE-OM. (XLSX 19 kb)

## References

[1] Tyler R, Baker L (1983). Difficulties experienced by tinnitus sufferers. J Speech Hear Disord.

[2] Loprinzi PD, Maskalick S, Brown K, Gilham B (2013). Association between depression and tinnitus in a nationally representative sample of US older adults. Aging Ment Health.

[3] Shargorodsky J, Curhan GC, Farwell WR (2010). Prevalence and characteristics of tinnitus among US adults. Am J Med.

[4] Michikawa T, Nishiwaki Y, Kikuchi Y, Saito H, Mizutari K, Okamoto M (2010). Prevalence and factors associated with tinnitus: a community-based study of Japanese elders. J Epidemiol.

[5] Langenbach M, Olderog M, Michel M, Albus C, Kohle K (2005). Psychosocial ad personality predictors of tinnitus related distress. Gen Hosp Psychiatry.

[6] Wallhausser-Franke E, Brade J, Balkenhol T, D'Amelio R, Seegmuller A, Delb W. Tinnitus: Distinguishing between Subjectively Perceived Loudness and Tinnitus-Related Distress. Plos One. 2012;7(4). doi:10.1371/journal.pone.0034583. 10.1371/journal.pone.0034583 PMC3329489 22529921

[7] McKenna L, Handscomb L, Hoare D, Hall D. A scientific cognitive behavioral model of tinnitus: novel conceptualizations of tinnitus distress. Frontiers in Neurology. 2014;5. doi:10.3389/fneur.2014.00196 10.3389/fneur.2014.00196 PMC4186305 25339938

[8] Conrad I, Kleinstauber M, Jasper K, Hiller W, Andersson G, Weise C (2015). The role of dysfunctional cognitions in patients with chronic tinnitus. Ear Hear.

[9] Probst T, Pryss R, Langguth B, Schlee W (2016). Emotional states as mediators between tinnitus loudness and tinnitus distress in daily life: Results from the “TrackYourTinnitus” application. Sci Report.

[10] Hiller W, Goebel G (2007). When tinnitus loudness and annoyance are discrepant: Audiological characteristics and psychological profile. Audiol Neuro Otol.

[11] Tunkel DE, Bauer CA, Sun GH, Rosenfeld RM, Chandrasekhar SS, Cunningham ER (2014). Clinical Practice Guideline: Tinnitus. Otolaryngol Head Neck Surg.

[12] Conrad I, Kleinstaeuber M, Jasper K, Hiller W, Andersson G, Weise C (2015). The changeability and predictive value of dysfunctional cognitions in cognitive behavior therapy for chronic tinnitus. Int J Behav Med.

[13] Hesser H, Gustafsson T, Lunden C, Henrikson O, Fattahi K, Johnsson E (2012). A randomized controlled trial of internet-delivered cognitive behavior therapy and acceptance and commitment therapy in the treatment of tinnitus. J Consult Clin Psychol.

[14] Robinson SK, Viirre ES, Bailey KA, Kindermann S, Minassian AL, Goldin PR (2008). A randomized controlled trial of cognitive-behavior therapy for tinnitus. Int Tinnitus J.

[15] Cima RFF, Maes IH, Joore MA, Scheyen D, El Refaie A, Baguley DM (2012). Specialised treatment based on cognitive behaviour therapy versus usual care for tinnitus: a randomised controlled trial. Lancet.

[16] Weise C, Heinecke K, Rief W (2008). Biofeedback- based behavioural treatment for chronic tinnitus: results of a randomized controlled trial. J Consult Clin Psychol.

[17] Kaldo V, Levin S, Widarsson J, Buhrman M, Larsen HC, Andersson G (2008). Internet versus group cognitive-behavioral treatment of distress associated with tinnitus: a randomized controlled trial. Behav Ther.

[18] Zigmond AS, Snaith RP. THE HOSPITAL ANXIETY AND DEPRESSION SCALE. Acta Psychiatrica Scandinavica. 1983;67(6):361–70. doi:10.1111/j.1600-0447.1983.tb09716.x 10.1111/j.1600-0447.1983.tb09716.x 6880820

[19] Beck AT, Erbaugh J, Ward CH, Mock J, Mendelsohn M (1961). An inventory for measuring depression. Arch Gen Psychiatry.

[20] Beck AT, Brown G, Epstein N, Steer RA (1988). An inventory for measuring clinical anxiety - psychometric properties. J Consult Clin Psychol.

[21] Zoger S, Svedlund J, Holgers KM (2001). Psychiatric disorders in tinnitus patients without severe hearing impairment: 24 month follow-up of patients at an audiological clinic. Audiology.

[22] Goebel G, Floetzinger U (2008). Pilot study to evaluate psychiatric co- morbidity in tinnitus patients with and without hyperacusis. Audiological Med.

[23] Evans C, Mellor-Clark J, Margison F, Barkham M, Audin K, Connell J (2000). CORE: Clinical Outcomes in Routine Evaluation. J Ment Health.

[24] Evans C, Connell J, Barkham M, Margison F, McGrath G, Mellor-Clark J (2002). Towards a standardised brief outcome measure: psychometric properties and utility of the CORE-OM. Br J Psychiatry.

[25] Uji M, Sakamoto A, Adachi K, Kitamura T (2012). Psychometnc properties of the Japanese version of the Clinical Outcomes in Routine Evaluation-Outcome Measure. Compr Psychiatry.

[26] Nordgren LB, Hedman E, Etienne J, Bodin J, Kadowaki A, Eriksson S (2014). Effectiveness and cost-effectiveness of individually tailored Internet-delivered cognitive behavior therapy for anxiety disorders in a primary care population: A randomized controlled trial. Behav Res Ther.

[27] Gilbody S, Richards D, Barkham M (2007). Diagnosing depression in primary care using self-completed instruments: UK validation of PHQ-9 and CORE-OM. Br J Gen Pract.

[28] Turner H, Marshall E, Stopa L, Waller G (2015). Cognitive-behavioural therapy for outpatients with eating disorders: Effectiveness for a transdiagnostic group in a routine clinical setting. Behav Res Ther.

[29] Barkham M, Culverwell A, Spindler K, Twigg E (2005). The CORE-OM in an older adult population: psychometric status, acceptability, and feasibility. Aging Ment Health.

[30] Bedford A, Watson R, Lyne J, Tibbles J, Davies F, Deary IJ (2010). Mokken Scaling and Principal Components Analyses of the CORE-OM in a Large Clinical Sample. Clin Psychol Psychother.

[31] Murray AL, McKenzie K, Murray KR, Richelieu M (2014). Mokken scales for testing both pre- and postintervention: an analysis of the Clinical Outcomes in Routine Evaluation-Outcome Measure (CORE-OM) before and after counseling. Psychol Assess.

[32] Lyne KJ, Barrett P, Evans C, Barkham M (2006). Dimensions of variation on the CORE-OM. Br J Clin Psychol.

[33] Skre I, Friborg O, Elgaroy S, Evans C, Myklebust LH, Lillevoll K (2013). The factor structure and psychometric properties of the Clinical Outcomes in Routine Evaluation--Outcome Measure (CORE-OM) in Norwegian clinical and non-clinical samples. BMC psychiatry.

[34] Handscomb L, Hall DA, Shorter G, Hoare DJ. Online data collection to evaluate a theoretical cognitive model of tinnitus. American Journal of Audiology. in press. 10.1044/2016_AJA-16-0007 PMC7617953 27768195

[35] Zeman F, Koller M, Schecklmann M, Langguth B, Landgrebe M, Grp TRIDS (2012). Tinnitus assessment by means of standardized self-report questionnaires: Psychometric properties of the Tinnitus Questionnaire (TQ), the Tinnitus Handicap Inventory (THI), and their short versions in an international and multi-lingual sample. Health Qual. Life Outcomes.

[36] Barkham M, Margison F, Leach C, Lucock M, Mellor-Clark J, Evans C (2001). Service profiling and outcomes benchmarking using the CORE-OM: Toward practice-based evidence in the psychological therapies. J Consult Clin Psychol.

[37] Steiger JH (1990). Structural Model Evaluation And Modification - An Interval Estimation Approach. Multivar Behav Res.

[38] Joreskog K, Sorbom D (1993). Structural Equation Modelling with the SIMPLIS command language.

[39] MacCallum RC, Browne MW, Sugawara HM (1996). Power analysis and determination of sample size for covariance structure modeling. Psychol Methods.

[40] Byrne B (2012). Structural equation Modelling with MPlus.

[41] Bentler PM (1990). Comparative Fit indexes in structural models. Psychol Bull.

[42] Tucker LR, Lewis C (1973). Reliability coefficient for maximum likelihood factor-analysis. Psychometrika.

[43] Hu L-t, Bentler PM (1999). Cutoff criteria for Fit indexes in covariance structure analysis: conventional criteria versus New alternatives. Struct Equ Modeling.

[44] Wilson PH, Henry J, Bowen M, Haralambous G (1991). Tinnitus reaction questionnaire - psychometric properties of a measure of distress associated with tinnitus. J Speech Hear Res.

[45] Meikle MB, Henry JA, Griest SE, Stewart BJ, Abrams HB, McArdle R (2012). The tinnitus functional index: development of a New clinical measure for chronic, intrusive tinnitus (vol 33, pg 153, 2012). Ear Hear.

[46] Spink M, Magrill A, Burgess K, Rogers J, Agius M (2014). Petals: an assessment of the outcomes of a service for bereavement during childbirth. Psychiatr Danub.

[47] Pridmore S, Walter G, Friedland P (2012). Tinnitus and suicide: recent cases on the public record give cause for reconsideration. Otolaryngol Head Neck Surg.

[48] Podsakoff PM, MacKenzie SB, Lee JY, Podsakoff NP (2003). Common method biases in behavioral research: A critical review of the literature and recommended remedies. J Appl Psychol.

[49] Vautier S, Pohl S (2009). Do balanced scales assess bipolar constructs? the case of the STAI scales. Psychol Assess.

