# Erratum to: Confirmatory factor analysis of Clinical Outcomes in Routine Evaluation (CORE-OM) used as a measure of emotional distress in people with tinnitus

**DOI:** 10.1186/s12955-017-0668-y

**Published:** 2017-05-03

**Authors:** L. Handscomb, D. A. Hall, D. J. Hoare, G. W. Shorter

**Affiliations:** 1 National Institute for Health Research Nottingham Hearing Biomedical Research Unit, Otology and Hearing Group, Division of Clinical Neuroscience, University of Nottingham, 113 The Ropewalk, Nottingham, NG1 5DU UK; 2 UCL Ear Institute, 332 Gray’s Inn Road, London, WC1X 8EE UK; 3 Alcohol and Public Health Team, Health and Social Care Institute, Teesside University, Middlesbrough, TS1 3BA UK; 4 Northern Ireland Association for Mental Health, 80 University St, Belfast, BT7 1HE UK; 5 Psychotraumatology, Mental Health, and Suicidal Behaviour Group, Psychology Research Institute, Ulster University, Coleraine, BT52 1SA UK

## Erratum

There was an inadvertent error in the original published article [1]. One part of the published ‘Results’ section (pp. 6] came from an older version of our manuscript, and the wording is not congruent with the published results table (Table S1).

The correct wording to describe Table S1 is as follows, but this does not affect the conclusion of the article:

“Model II comprising negative, positive and risk factors provided a fairly good fit. In Model III, omitting items 6 and 22 from the risk factor resulted in a slightly poorer fit. Model IV, in which five items were deleted and four moved from the negative factor to the risk factor was a comparable fit to Model II. Model V, the two factor structure recommended by Skre et al. ([33] Reference [2] here) yielded a reasonable fit. In model VI, omitting items 6 and 22 from the risk factor resulted in a poorer fit. Model VII, the 28-item, two factor model, was also a worse fit than Model V. Model VIII, the single-factor model including all items, did not meet good fit criteria. Model IX, the shortened 6-item scale, was a mediocre fit, while model X, the 10-item scale, was an acceptable fit to the data. Model II was chosen as the optimal model as it was a good fit to the data and closer to the original questionnaire than Model IV. For all items in Model II, factor loadings were positive, moderately to very high and statistically significant. There was a high degree of positive correlation between all three factors.

Factor scores for each of the three factors in the optimal model (Model II) are shown in Table 3, overall and divided by tinnitus problem category.”

This should replace the following:

“Model II comprising negative, positive and risk factors provided the best fit. In Model III, omitting items 6 and 22 from the risk factor resulted in a much poorer fit. Model IV, in which the items were differently distributed between the negative, positive and risk factors, was a very poor fit. Model V, the two factor structure recommended by Skre et al. ([33] Reference [2] here) yielded a reasonable fit. In model VI, omitting items 6 and 22 from the risk factor again resulted in a poorer fit. Model VII, the 28-item, two factor model, was also a worse fit than Model V. Model VIII, the single-factor model including all items, did not meet good fit criteria. Model IX, the shortened 6-item scale, was a mediocre fit, while model X, the 10-item scale, was an acceptable fit to the data. The best fitting model overall was model II. For all items, factor loadings were positive, moderately to very high and statistically significant. There was a high degree of positive correlation between all three factors.

Factor scores for each of the three factors in the best fitting model (Model II) are shown in Table 3, overall and divided by tinnitus problem category.”

## References

[1] Handscomb L, Hall DA, Hoare DJ, Shorter GW (2016). Confirmatory factor analysis of Clinical Outcomes in Routine Evaluation (CORE-OM) used as a measure of emotional distress in people with tinnitus. Health Qual Life Outcomes.

[2] Skre I, Friborg O, Elgaroy S, Evans C, Myklebust LH, Lillevoll K (2013). The factor structure and psychometric properties of the Clinical Outcomes in Routine Evaluation--Outcome Measure (CORE-OM) in Norwegian clinical and non-clinical samples. BMC Psychiatry.

